# CT-TEE Image Registration for Surgical Navigation of Congenital Heart Disease Based on a Cycle Adversarial Network

**DOI:** 10.1155/2020/4942121

**Published:** 2020-07-02

**Authors:** Yunfei Lu, Bing Li, Ningtao Liu, Jia-Wei Chen, Li Xiao, Shuiping Gou, Linlin Chen, Meiping Huang, Jian Zhuang

**Affiliations:** ^1^Key Laboratory of Intelligent Perception and Image Understanding of Ministry of Education, School of Artificial Intelligence, Xidian University, Xi'an 710071, China; ^2^Department of Infectious Diseases, Ankang Central Hospital, Ankang 725000, China; ^3^Institute of Computing Technology, Chinese Academy of Sciences, Beijing 100190, China; ^4^Catheterization Lab, Guangdong Cardiovascular Institute, Guangdong Provincial Key Laboratory of South China, Structural Heart Disease, Guangdong General Hospital, Guangdong Academy of Medical Sciences, Guangzhou 510000, China; ^5^Department of Cardiac Surgery, Structural Heart Disease, Guangdong General Hospital, Guangzhou 510000, China

## Abstract

Transesophageal echocardiography (TEE) has become an essential tool in interventional cardiologist's daily toolbox which allows a continuous visualization of the movement of the visceral organ without trauma and the observation of the heartbeat in real time, due to the sensor's location at the esophagus directly behind the heart and it becomes useful for navigation during the surgery. However, TEE images provide very limited data on clear anatomically cardiac structures. Instead, computed tomography (CT) images can provide anatomical information of cardiac structures, which can be used as guidance to interpret TEE images. In this paper, we will focus on how to transfer the anatomical information from CT images to TEE images via registration, which is quite challenging but significant to physicians and clinicians due to the extreme morphological deformation and different appearance between CT and TEE images of the same person. In this paper, we proposed a learning-based method to register cardiac CT images to TEE images. In the proposed method, to reduce the deformation between two images, we introduce the Cycle Generative Adversarial Network (CycleGAN) into our method simulating TEE-like images from CT images to reduce their appearance gap. Then, we perform nongrid registration to align TEE-like images with TEE images. The experimental results on both children' and adults' CT and TEE images show that our proposed method outperforms other compared methods. It is quite noted that reducing the appearance gap between CT and TEE images can benefit physicians and clinicians to get the anatomical information of ROIs in TEE images during the cardiac surgical operation.

## 1. Introduction

Congenital heart disease accounts for 28% of all congenital malformations. In China, the incidence of congenital heart disease is 0.4-1% among the infants approximately 150000-200000 newborns annually. Transesophageal echocardiography (TEE) is an imaging of congenital heart disease. The sensor is usually placed at the esophagus directly behind the heart and allows a continuous visualization of the movement of the visceral organ without trauma and the observation of the heartbeat in real time. Therefore, it has become an essential tool for most interventional cardiologists in navigation during the surgery. However, TEE images usually provide very limited data on clear anatomically cardiac structures, which makes TEE images difficult to interpret. While high-resolution computed tomography (CT) images can provide anatomical information of cardiac structures, a CT scanner is not flexible to move and cannot be used in surgery. Despite the limitations of a CT scanner, the sufficient anatomical information of organs in CT images can benefit us to transfer the anatomical information to TEE images by the registrations between CT and TEE images.

Recently, similar ideas have been proposed to transfer the anatomical information of prostate from magnetic resonance (MR) image to CT images via registration methods. Cao et al. [1] developed a bidirection registration method from MR images to CT images via simulating CT and MR images with a structural random forest. Fundamentally, the similarity measurement is a core issue in registration. Recently, Cao et al. [2] proposed a similarity measurement for a registration-based convolution neural network. Fan et al. [3] proposed a registration method-based adversarial similarity network.

However, the registration between CT and TEE images is more challenging. For one, the quality of TEE images is much lower than other modalities, especially for children' cardiac TEE images. For others, the appearance patterns of CT and TEE images are quite different, even from the same subject. This big gap on appearance between two images makes most typical nongrid registration methods [4] be inefficient. It is quite significant in surgical operation to reduce the appearance gaps between these two modalities of medical images.

To reduce the appearance gap cross-image modalities, a generative adversarial network (GAN) [5] has been proposed to generate an image following a distribution. Nie et al. [6] introduced the GAN in medical image synthesis. Tanner et al. [7] proposed a GAN for MR-CT deformable registration. Yan et al. [8] proposed an end-to-end adversarial network for MR and TRUS image fusion. However, most GANs need paired data to train and it is difficult to collect sufficient number of paired medical image data. Wolterink et al. [9] proposed a GAN to synthesize CT image from an MR image which is trained by unpaired image data. On the other hand, Zhu et al. [10] proposed the Cycle Generative Adversarial Network (CycleGAN) for the image translation from different domains. Compared with the GAN, it can be efficiently trained by the unpaired image data, which are more easily collected. This advantage can benefit to the cross-domain medical image registration. In particular, for the cross-modal medical images with big appearance and morphological gaps, CycleGAN can be introduced to them.

Inspired by this, in this paper, we proposed a learning-based registration method to align CT and TEE images of the same subject. In the proposed method, we introduce a cycle adversarial network to generate TEE-like images from the corresponding CT images and CT-like images from the corresponding TEE images, with which reduces the appearance gap between two modalities. After that, the nongrid registration methods are applied to align TEE-like images with TEE images and CT images with CT-like images, respectively, to obtain two deformation fields. The final registration results can obtained by averaging these two deformation fields. It is quite significant to reduce the appearance gap between CT and TEE images which can benefit physicians and clinicians to get the anatomical information of ROIs in TEE images during the cardiac surgical operation.

The rest of this paper can be organized as follows. The proposed method will be introduced in Section 2, and the experimental results will be presented in Section 3. Finally, the conclusion will be made in Section 4.

## 2. Method

### 2.1. Datasets and Preprocessing

In this study, we collect a dataset of paired CT images and TEE images of 12 subjects with congenital heart disease. They include 2 adults and 10 teenagers. All the CT images are scanned by the SIEMENS CT VA1 DUMMY scanner, and their sizes of the *XoY* planes are 512 × 512. Their spacing variously ranges from 0.28 × 0.28 mm^2^ to 0.45 × 0.45 mm^2^, while the TEE images are scanned by Philips iE33 Medical Imaging System with the size of the *XoY* plane being 800 × 600. Their spacing variously ranges from 0.15 × 0.15 mm ^2^ to 0.28 × 0.28 mm ^2^. Due to the difficulty of data acquisition, TEE images for all patients only include 22 standard sections of the heart. Before training the CycleGAN, we resample all the images into the same spacing 0.45 × 0.45 mm^2^ and crop all the image into the same size.

### 2.2. CT-TEE Image Generation by CycleGAN

**Figure 1:**
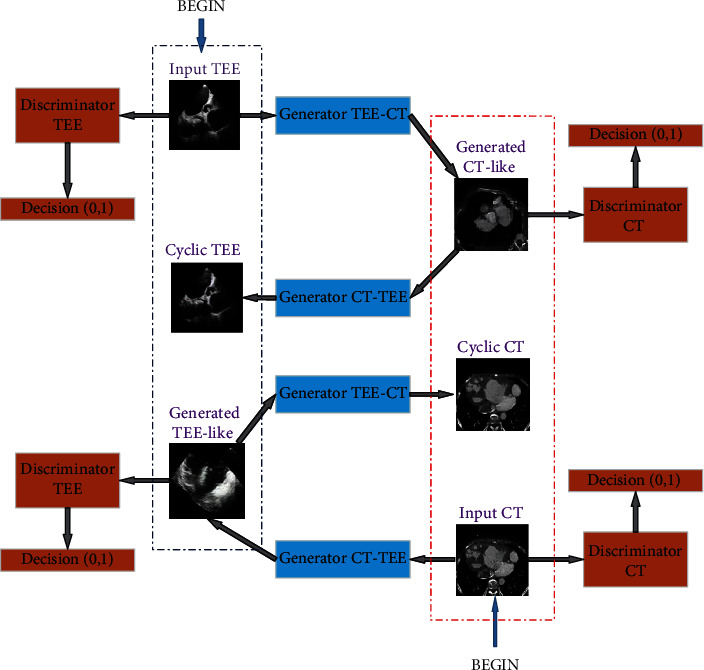
The architecture of CT-TEE image generation.

The CycleGAN is a weakly supervised GAN that is trained by unpaired samples achieving the cross-domain image translation with different distributions. In this section, we introduce it to CT-TEE image generation to reduce the appearance and morphological gap between them. The architecture of CT-TEE image generation with CycleGAN can be illustrated in Figure 1.

As shown in Figure 1, the architecture contains two directions of image generation: from a TEE image to generate a CT-like image and from a CT image to generate a TEE-like image. First, starting a TEE image as illustrated the top-left “BEGIN” in the figure, a CT-like image can be generated by the TEE-CT generator and then, a cyclic TEE image will be generated by the CT-TEE generator. The generated cyclic TEE image is forwarded to a discriminator giving a measure how it is like the real TEE image.

On the other thread, starting a CT image as illustrated the bottom-right “BEGIN” in the figure, a TEE-like image can be generated by the CT-TEE generator and then, a cyclic CT image will be generated by the TEE-CT generator. Similarly, the generator cyclic CT image is forwarded to a discriminator to measure how it is like the real CT image. The generator here is designed as the one in [11].

**Figure 2:**
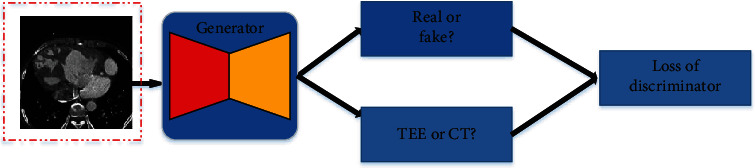
An illustration of discriminator.

The discriminator here can be illustrated in Figure 2. It is shown that the discriminator includes two parts. The one is used to determine whether the generated image is a real or fake, and the other is used to classify whether the generated image is a TEE or a CT image. The discriminative loss will be used to update the parameters of the whole network. It can be computed as follows:

(1) $$\begin{array}{c} L_{GAN}\left(G,D_{1},D_{2},X,Y\right)=E_{y∼P\left(Y\right)}\left[{\left(D_{1}\left(y\right)-1\right)}^{2}\right]+E_{y∼P\left(Y\right)}\left[D_{1}{\left(G\left(x\right)\right)}^{2}\right]+\alpha \cdot \left(E_{y∼P\left(Y\right)}\left[{\left(D_{2}\left(y\right)-1\right)}^{2}\right]+E_{y∼P\left(Y\right)}\left[D_{2}{\left(G\left(x\right)\right)}^{2}\right]\right), \end{array}$$

where *G* and *D*_1_, *D*_2_ denote the generator and discriminator, respectively. *X* and *Y* denote the CT and TEE domains, respectively. *α* is a weight which balances two parts of discriminative loss.

### 2.3. CT-TEE Registration Based on Generated Images

**Figure 3:**
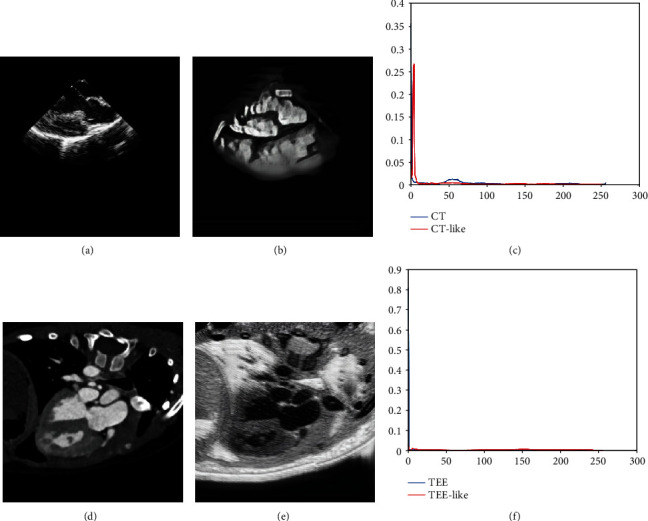
Comparisons between original images and generated images. (a) Original TEE image. (b) CT-like image. (c) Histograms of TEE image and CT-like image. (d) Original CT image. (e) TEE-like image. (f) Histograms of CT image and TEE-like image.

It is a great challenge to perform CT-TEE registration due to the appearance and morphological gaps between CT and TEE images. TEE and CT images are shown in Figures 3(a) and 3(d), respectively. It can be observed that the cardiac in TEE and CT images appears to have a different appearance and morphology. In the above paragraphs, we have introduced the CycleGAN to perform CT-TEE image translation, reducing the appearance and morphological gaps.

**Figure 4:**
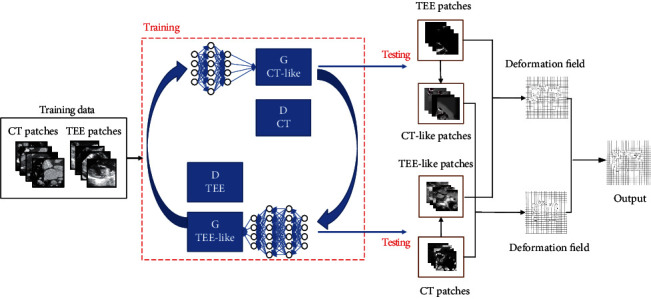
The whole framework of the proposed method.

In this section, we will propose a learning-based registration method to reduce the appearance gap between TEE images and CT images by introducing a cycle adversarial network (CycleGAN). The whole framework can be illustrated by Figure 4.

In this framework, we first generate a TEE-like image from the corresponding CT image, and a CT-like image from the corresponding TEE image with the trained CycleGAN. By this network, the generated TEE-like images with the same morphological feature with the corresponding CT images of the same subject possess the similar appearance features to TEE images, while the generated CT-like images with the same morphological features with the corresponding TEE images, possess the similar appearance features to CT images.

Then, we can perform the registration between CT images and TEE images through aligning TEE-like images with TEE images and CT images with CT-like images, respectively.

### 2.4. Training Network

**Figure 5:**
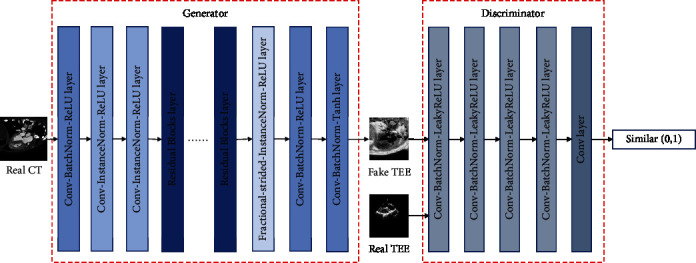
The network structure of CycleGAN.

The structure of the adversarial network we employ in this paper is illustrated in Figure 5. In this network, we employ a convolution network with 7 convolution-BatchNorm-ReLU layers as the generator. On the other hand, we introduce another convolution network with 5 Convolution-BatchNorm-ReLU layers as the discriminator.

To train CycleGANs between CT images and TEE images, we randomly draw patches with size 256 × 256 from the collected dataset, where 9750 and 15020 ones are collected from CT images and TEE images, respectively. The model is trained on a PC with Intel i7 9700K CPU, 64GB memory and NVIDIA TITAN Xp GPU. The platform is built on TensorFlow with Ubuntu 16.04. In the training process, the network is optimized by the Adam algorithm, where the batchsize and learning rate are set as 2 and 2 × 10^−4^, respectively. The training loss becomes stable after 19055 iterations.

## 3. Experiment Results

In the following experiments, the proposed model is verified by the leave-one-out validation; i.e., a patient is taken out first as a testing subject and the rest subjects are employed to train the CycleGAN.

### 3.1. Image Generation Results

The comparisons between the original image and generated image are shown in Figure 3.The results show that the CT-like images generated from TEE images possess not only the same morphologic structure as original TEE images but also the similar appearance feature with CT images. The TEE-like image and TEE image tell the same story. Furthermore, the histogram in Figures 3(c) and 3(f) shows that the intensity distribution of the CT-like image is quite similar to the CT image, while the TEE-like image follows the similar distribution to the TEE image.

**Figure 6:**
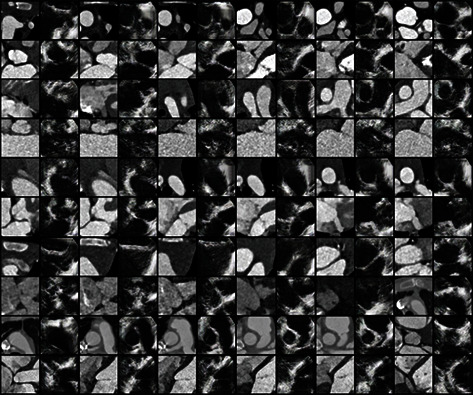
The TEE-like images generated from CT images.

More TEE-like images are shown in Figure 6, where CT images are shown in odd columns and the corresponding TEE-like images are shown in even columns. It can be observed from numerous results that the framework in Figure 1 can reduce the gap between CT and TEE images which provides a good precondition for the following registration.

### 3.2. Image Registration Results

In the registration process, we employ the FLIRT [12] method as grid registration followed by Powell and Demons [4] as nongrid registration methods. Through the deformation field, we map the labels from CT images with the labels in TEE images of the same organ.

**Figure 7:**
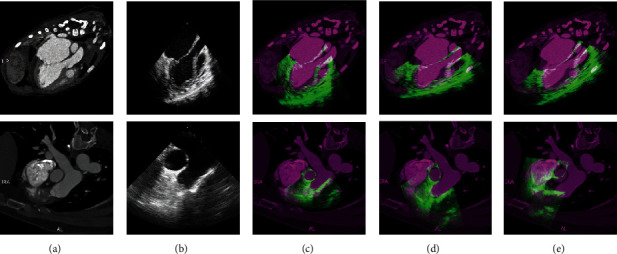
The registration result. (a) Original CT image. (b) Original TEE image. Results of (c) the proposed method. (d) FLIRT. (e) Demons registration.

We show the registration results of two subjects in Figure 7. We applied FLIRT and Demons registrations to perform registration between CT and TEE images and overlapped the CT image and the aligned TEE image. The results are shown in the panel (d) and (e). We can observe that two images are not well-aligned in both two subjects due to their appearance and morphological gaps. Instead, through CycleGAN, we perform the registration between the CT-like image and the CT image. It is shown in the panel (c) that we can get better alignment performance.

**Figure 8:**
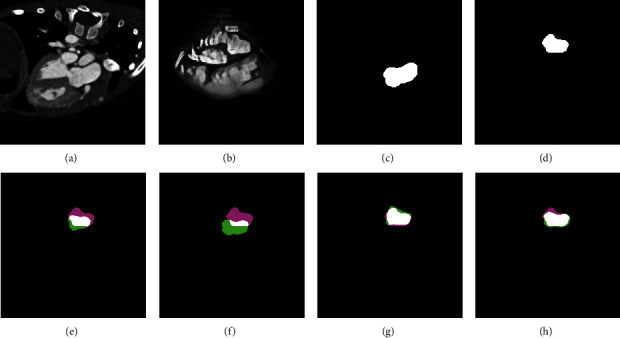
The registration of an adult subject. (a) Original CT image. (b) CT-like image. (c) Ground truth on CT domain. (d) Ground truth on TEE domain. (e) Demons registration on original image. (f) Powell registration on original image. (g) Demons registration on generated image. (h) Powell registration on generated image.

**Figure 9:**
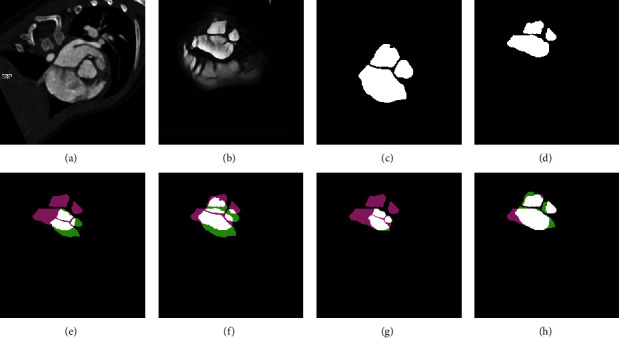
The registration of a teenager subject. (a) Original CT image. (b) CT-like image. (c) Ground truth on CT domain. (d) Ground truth on TEE domain. (e) Demons registration on original image. (f) Powell registration on original image. (g) Demons registration on generated image. (h) Powell registration on generated image.

We also show another two examples including an adult subject and a teenager subject of registration results in Figures 8 and 9, respectively, where in the panels (e)-(h), the pink indicates the ground truth of TEE domain, the green indicates the mapped labels from CT domain to TEE domain, and the white indicates the overlap of them. It can be shown from Figures 8(e) and 8(f) that the overlapping regions in Figures 8(g) and 8(h) are larger than (e) and (f), respectively. It means that the better alignments are obtained on generated images than original images. Similar visual results can also be obtained on the teenager subject. Our proposed method can tackle with both adult subjects and teenager subjects.

Furthermore, the registration performances are evaluated by the Dice ratio (DR), Hausdorff distance with percentile of 95% (HD95) [13] and the average symmetric surface distance(ASD) between the mapped labels through the deformation field and the ground truth in TEE domain. Hausdorff distance with percentile of 95% (95% HD) is based on the calculation of the 95th percentile of the Hausdorff distance between boundary points in two sets, Hausdorff distance means the maximum value of the shortest distance from a point set to another point set. Better results can get higher DR and lower HD95 and ASD. We show the mapped labels from the CT image and the ground truth on the TEE image, where we compare the Demons and Powell methods on both original images (OI) and generated images (GI). We list the means and standard deviations of evaluations on both teenager and adult registration results in Table 1. In terms of Dice Ratio, the values on the GI are much larger than the ones on the original images, while in terms of HD95 and ASD, the registrations on GI get lower ones than those on OI. It is demonstrated that classic nongrid registration methods are almost noneffective on original CT and TEE images alignment. Instead, the performances of registrations on generated images improve significantly than original images.

It is demonstrated from all of the above visual results and quantitative evaluations that our proposed method is effective for CT-TEE registration. It is benefited from the CycleGAN that reduces the appearance gaps between CT and TEE images. However, Demons is a nonrigid registration method that is easy to fall into local optimum, and the registration result is usually not good when there is a certain difference in shape and size between two images, panels (g) and (h) in Figure 9 show the limitation of Demons.

## 4. Conclusion

It is quite significant to reduce the appearance gap between CT and TEE images which can benefit physicians and clinicians to get the anatomical information of ROIs in TEE images during the cardiac surgical operation. In this paper, we develop a CycleGAN-based registration method to align CT images with TEE images. The CycleGAN reduces the appearance gap between CT images and TEE images. Our proposed method is verified on 12 pairs of CT-TEE images. Both visual results and quantitative evaluations show that the performance of the registration with generated images is better than original images. It indicates that our proposed method can get reasonable registration results between CT and TEE images with the challenges of large appearance.

## Figures and Tables

**Table 1:** Quantitative evaluations of registrations.

	DR	HD95	ASD
Demons on OI	0.31 ± 0.22	76.50 ± 52.17	32.91 ± 37.98
Demons on GI	0.75 ± 0.09	33.83 ± 16.17	9.83 ± 3.75
Powell on OI	0.26 ± 0.29	84.81 ± 48.41	40.69 ± 28.38
Powell on GI	0.78 ± 0.05	32.16 ± 16.89	8.61 ± 4.62

## Data Availability

The data used in the article are from the Department of Cardiac Surgery, Structural Heart Disease, Guangdong General Hospital, Guangzhou, China.
